# One data set, many analysts: Implications for practicing scientists

**DOI:** 10.3389/fpsyg.2023.1094150

**Published:** 2023-02-14

**Authors:** Erich Kummerfeld, Galin L. Jones

**Affiliations:** ^1^Institute for Health Informatics, University of Minnesota, Minneapolis, MN, United States; ^2^School of Statistics, University of Minnesota, Minneapolis, MN, United States

**Keywords:** reproducibility, data analysis, metascience, multilab analysis, statistical problem-solving process

## Abstract

Researchers routinely face choices throughout the data analysis process. It is often opaque to readers how these choices are made, how they affect the findings, and whether or not data analysis results are unduly influenced by subjective decisions. This concern is spurring numerous investigations into the variability of data analysis results. The findings demonstrate that different teams analyzing the same data may reach different conclusions. This is the “many-analysts” problem. Previous research on the many-analysts problem focused on demonstrating its existence, without identifying specific practices for solving it. We address this gap by identifying three pitfalls that have contributed to the variability observed in many-analysts publications and providing suggestions on how to avoid them.

## 1. Introduction

Researchers face choices throughout the data analysis process. It is often opaque how these choices are made, and how they affect the results. Even assuming good-intent (e.g., no p-hacking or fraud), how do we know when data analysis results are not unduly the result of arbitrary, subjective decisions? This concern is spurring numerous investigations into the variability of data analysis across different teams of analysts (Silberzahn et al., 2018; van Dongen et al., 2019; Barcus et al., 2020; Botvinik-Nezer et al., 2020; Landy et al., 2020; Ney et al., 2020; Breznau et al., 2021; Hoffmann et al., 2021). This work has demonstrated that different teams analyzing the same data may reach different conclusions. This is the “many-analysts” problem. Due to the importance of data analysis, the observed variability in many-analysts papers initiated a burgeoning research area, including a roadmap for conducting future many-analysts studies (Aczel et al., 2021).

Many-analysts publications have focused on giving multiple teams the same problem and evaluating the variability of their results. Typically they do not address the causes of the observed variability, or constructive ways of controlling it. This is addressed here by synthesizing existing research and identifying three common pitfalls with practical solutions for avoiding them.

Many-analysts publications emphasize the presence of seemingly unavoidable “subjectivity.” For example, “The observed results from analyzing a complex data set can be highly contingent on justifiable, but subjective, analytic decisions'' (Silberzahn et al., 2018). Additionally, Aczel et al. (2021) emphasize the same point “...empirical results typically hinge on analytical choices made by just one or a small number of researchers, and raises the possibility that different—perhaps equally justifiable—analytical choices may produce different results.” Often the proposed solution is transparency: “The best defense against subjectivity in science is to expose it” (Silberzahn et al., 2018). While transparency is important, and “subjectivity” does play some role in creating variability, researchers can do better than simply monitor their activities in more detail: they can adopt improved research practices.

The variability found in the many-analysts projects is largely explained by concrete and modifiable elements of their design. This is especially so for some of the earliest elements of the data analysis process that many projects did not, but could have, controlled. This includes clearly identifying the problem to be solved, and building the analysis team. This position is supported with a detailed review of these projects, including some having little variability among their teams' results. To their credit, often the many-analysts projects operate according to the philosophy of open science, in line with their recommendations. This ultimately shed light on the topic, helped form the position put forward here, and provided substantial evidence for it.

## 2. Real world data science

**Figure 1:**
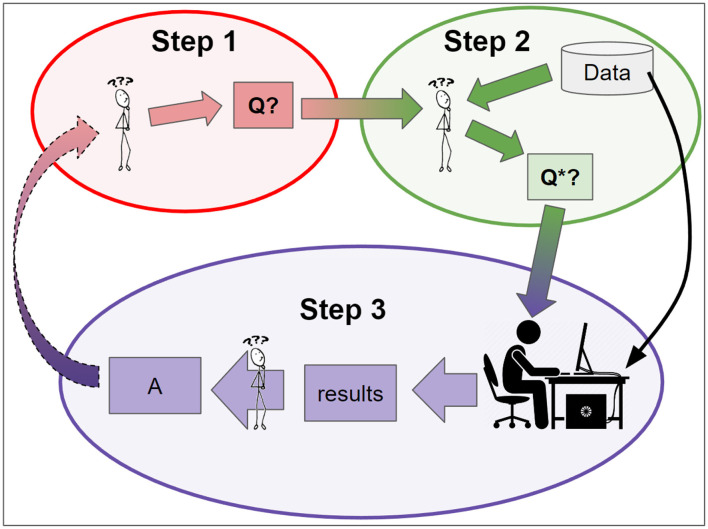
A conceptual model of real world data analysis and its role within the larger process of inquiry. Step 1: the investigators identify a big picture question: Q. Step 2: they refine Q into a mathematically precise question, Q*, that can be directly addressed with available data and methods. Step 3: a formal data analysis process is applied to the data, and results are interpreted to produce an answer to Q* and improve understanding of Q. This answer may lead to the development or refinement of new big picture questions, thereby spurring further inquiry.

Investigations of the data analysis process often start by assuming that there is a specific data set to be analyzed and a corresponding well-specified technical question, such as determining whether one mathematically-defined quantity is greater than another. Real world data science projects, however, often spend a great deal of time simply getting to that point. For simplicity, the issue of selecting what data set to analyze is ignored, since it is a practical reality that many projects begin with the intention of analyzing a specific data set, with no intention to analyze other data sets. Determining a well-specified technical question, however, is something that must be addressed. In fact, many projects struggle prior to that, while trying to determine the big picture problems or questions that they want their analysis to address. Figure 1 presents a high-level view of a data analysis project, broken down into three main steps.

In the first step researchers identify Q, the question they want answered. Q typically employs non-technical terms that engage our human concerns more than technical definitions. For example, “Should we be using [Treatment X] for [Disease Y] or not?''. Many-analysts papers often call this the “hypothesis” being tested.

In the second step, the researcher considers Q, the available data, and the methods they can employ, in order to establish Q*, a mathematically precise question that (1) is as closely relevant to Q as possible, and (2) can be answered with available data and methods. The importance of this step has been noted previously (Seok et al., 2013; Takao and Miyakawa, 2015). Examples of Q* include:

1. Did patients with [Disease Y] who received [Treatment X] have lower 1-year mortality than those who did not, in our hospital's EHR data?
2. Across patients with [Disease Y], is the estimated causal effect of [Treatment X] on 1-year mortality rate in our hospital's EHR data—taking into consideration other factors such as demographics, ICU status, and disease severity—positive or negative, and by how much?

Various psychological factors, such as desire to use more data, fear of using variables or samples that the analyst is unfamiliar with, or expertise in statistical methodologies, will influence the Q*(s) ultimately pursued by any specific team. It is common for Step 2 to be repeated, as more than one Q* may be relevant to Q. This can occur both within and across research teams. Importantly, not all Q*s are as actionable with the available data and methods or as relevant to Q as others. We will discuss this more in Section 3.2.

In the third step, data analysis experts use available methods and data to answer Q*. Theoretical investigations of the data analysis process often emphasize this step, and ignore or suppress Steps 1 and 2. The many-analysts papers themselves often do this. For example,

There are three pitfalls that, when unresolved, apparently produce substantial variation in the results of the many-analysts papers. These pitfalls occur primarily in Steps 1 and 2:

1. Lack of an actionable overarching question.
2. Failure to explicitly identify a formal question using language from the data and methods.
3. Team lacks some relevant expertise.

Notably, we are aware of one many-analysts paper that avoided the pitfalls and did not find substantial variation among their results, lending credence to our claim (van Dongen et al., 2019).

## 3. Pitfalls and suggested solutions

Examples of the following pitfalls are common in many-analyst papers. We provide specific examples for each, (a) as demonstration that the pitfalls can co-occur and (b) to maintain focus on the pitfalls rather than attempting a comprehensive survey of many-analyst papers.

### 3.1. Pitfall 1: Lack of an actionable overarching question

A vague Q can lead to an unsound foundation for the entire project. An ideal Q is easy to understand and communicate, unifies the research team, and points toward specific research directions—i.e., Q*. Vague questions may be understood differently by different team members, and may leave researchers unsure how to proceed with data collection or analysis, or even what would constitute an acceptable answer. For example, Q may contain terms that are unclear or lack clarity about their definition.

Silberzahn et al. (2018) asked 29 analysis teams the same natural language question: “(Are) soccer referees more likely to give red cards to dark-skin-toned players than to light-skin-toned players?” On the face of it, the question seems straightforward, but it should be clarified prior to data collection and analysis. What population of soccer players is of interest? Should the analysis adjust for league, player position, referee experience, or referee skin-tone? Notice that dark-skin-toned vs. light-skin-toned may be defined differently in different contexts.

Silberzahn et al. (2018) provided a common data set to the teams. In the overarching question there was no mention of covariates, but the data had many. No guidance was given about which were of interest, or why they were included. The email correspondence between teams, which was published as part of the project, shows substantial disagreement among the teams regarding how to formalize the overarching question—in other words, how to develop Q*. One team wrote: “I think most ... of these variables”... should probably be included, but ultimately we can't be sure given the way the question was framed.” Another team wrote: “The question, as written, is ‘Are soccer referees more likely to give red cards to dark skin toned players than light skin toned players?' Of course I have assumed that the actual question of interest is whether dark skin toned players receive more red cards ceteris paribus, or, solely because they have darker skin and not because of something else.” Another team defended an opposing approach: “We did not use any covariates such as player position and decided to stick with this approach even though reviewers of our approach suggested that we should do so. As already noted in the project description ... the data cannot be used for causal inference. Thus, if the goal is to come up with a generalizable descriptive statement (i.e., effect size), it does not matter why a player ends up getting more red cards (e.g., being a tall, heavy defense player).”

Silberzahn et al. (2018) reported their primary conclusion as “...results from analyzing a complex data set can be highly contingent on justifiable, but subjective, *analytic* decisions.” [emphasis added] However, a substantial source of the observed variability is apparently simply that the different teams attempted to answer vastly different Q*s; the decision to include or ignore any particular covariate formally changes the question being addressed. This suggests that comparing their answers is akin to comparing apples and oranges.

In contrast, van Dongen et al. (2019) considered the impact of different statistical paradigms, namely Bayesian vs. frequentist, to determine “...does it matter?” However, for the two projects presented to their participating teams, they provided clear and simple Qs coupled with straightforward data sets. The first project asked “Is cetirizine exposure during pregnancy associated with a higher incidence of birth defects?” and in the second project, “Do PTSD patients with high resting state amygdalar activity experience more stress?” The data provided was well-suited to the questions and did not inject any confusion into the process. van Dongen et al. (2019) noted that “despite employing widely different approaches, all teams nevertheless arrived at a similar conclusion.”

#### Avoiding the pitfall

The initial question should be as specific and actionable as reasonably possible; see Hand (1994) and Hernán (2016) for more on specificity of research questions. It will pay dividends to acknowledge the vagueness or limitations of the overarching question prior to moving forward with the analysis. A few examples of things to check about a potential Q are:

1. Are the units of analysis clear for any Q* that addresses Q, i.e., is it clear whether the question is about people, places, groups, individuals, etc.?
2. Is it clear what kind of statement would answer this question? Can it be answered by a “yes,” or a number, or a map, or something else?
3. Is it clear what sort of context the question assumes? Does it only apply to a specific population, region, or species?
4. Are the terms used in the question well defined and agreed upon by the intended research community?
5. Does the question help identify what variables should be included for analysis?

### 3.2. Pitfall 2: Failure to identify a formal question using language from the data and methods

While Q is the initial overarching question, Q* is the formal (statistical) question that can be evaluated with the data. Even though different analysts can agree on the same Q, as mentioned in the previous section circumstances can lead them to addressing different Q*s. This can lead to seemingly different conclusions. It is crucial to understand the different Q*s being addressed to be able to evaluate their conclusions.

Sufficiently broad Qs may be translated into multiple Q*s that have apparently contradicting answers. This commonly leads to vigorous debate, which may be productive and is a normal part of inquiry. For example, Seok et al. (2013) and Takao and Miyakawa (2015) published papers with the striking titles of “Genomic responses in mouse models greatly mimic human inflammatory diseases” and “Genomic responses in mouse models poorly mimic human inflammatory diseases,” respectively. Ostensibly they reach opposite conclusions based on an analysis of the same data. However, a closer examination shows that Takao and Myakawa analyze only a subset of the variables analyzed by Seok et al. In our framework, the two papers address different Q*s, and the letters responding to these papers illustrate that it is open to debate whether and how these Q*s address the overarching question: “how well do genomic responses in mouse models mimic human inflammatory diseases?”

Landy et al. (2020) ask “To what extent are research results influenced by subjective decisions that scientists make as they design studies?” This has a close connection to what we call Step 2 in the data analysis process. In their study, teams of investigators designed their own studies to address a research hypothesis. The teams were blinded to the other teams' approaches and results, the researchers were constrained to constructing a short on-line questionnaire, and the statistical methods were constrained to be either a Pearson correlation or a simple test of differences. Landy et al. (2020) found “dramatic consequences of researcher design choices for scientific results.” In this project, a clear Q is provided, and the analysis methods used are restricted to be extremely simple. Pitfall 2, however, is purposefully left in play: the different teams are free to collect different kinds of data, and thus even the language used in their Q*s will differ, let alone their content. As such, this is an excellent demonstration of the potential impact that Pitfall 2 has on the variability of a project's results.

Importantly, this pitfall is *not* that there can be multiple viable Q*s. The pitfall arises when investigators are unclear about *what* their Q* is, what assumptions they are relying on to select this Q*, or the limitations of using this Q* to address Q.

#### Avoiding the pitfall

Explicitly acknowledge that the issue must be addressed. The team should produce a Q* that captures some agreement between Q, available data, and available methods. Developing a good Q* often requires substantial domain expertise, data expertise, and statistical expertise, to know both what questions are worth answering and what questions can be answered. When no singular Q* can directly address Q, researchers could consider using approaches such as triangulation, where multiple Q*'s are developed and addressed in parallel and evaluated collectively.

### 3.3. Pitfall 3: Team lacks some relevant expertise

Avoiding Pitfalls 1 and 2 require team members with the relevant expertise, whether it be in the topic, data, or analysis methods. Pitfall 3 applies throughout the project, from its conceptualization to its execution and reporting, and additional team members may need to be added during these phases, as new problems or confusions are encountered. Building a team that has expertise in all relevant areas can be difficult, and many projects lack critical expertise on their team. When expertise is lacking, mistakes or outright errors become far more likely, with unpredictable impact on the results.

**Figure 2:**
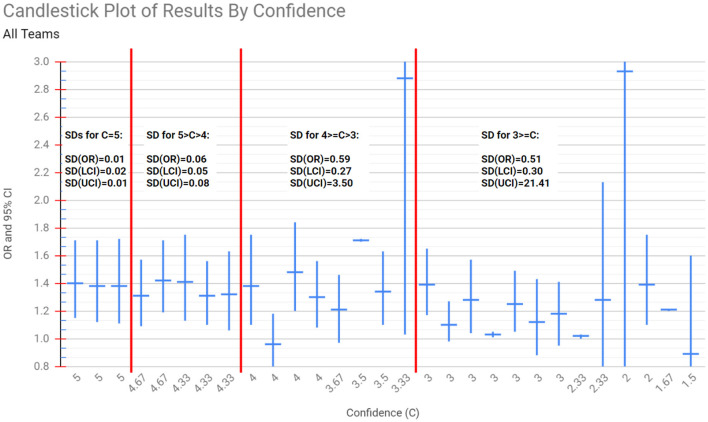
The ORs and 95% CIs reported by the analysis teams in Silberzahn et al. (2018), sorted from left to right in decreasing order of Confidence (C), as rated by their peers (a proxy for peer estimated expertise). The vertical red lines separate groups of teams whose Confidence fell into specific categories: *C* = 5, 5 > *C* > 4, 4 > = *C* > 3, and *C* <= 3. The variance among the ORs and CIs of the teams that received high Confidence scores is low, but increases as C decreases. For example, across the teams with *C* = 5, the standard deviation (SD) of their Odds Ratio (OR), Lower Confidence Interval (LCI) and Upper Confidence Interval (UCI) are 0.01, 0.02, and 0.01 respectively, while for teams with *C* <= 3 the corresponding SDs are 0.51, 0.30, and 21.41. This inverse relationship between Confidence and the variance of results across teams was not identified explicitly by Silberzahn et al. (2018).

Figure 2 displays the results of the 29 teams from Silberzahn et al. (2018). They are sorted by “Confidence,” a peer review numerical measure assessing the confidence their peer teams had in them after assessing their work, from highest (left) to lowest (right). Higher values correspond to more confidence. We are treating it as a proxy for expertise. Teams that received high confidence scores (high expertise) had more similar results, while teams receiving low confidence scores (low expertise) had more varied results.

Recall that van Dongen et al. (2019) did not observe the same variability in results observed by other many-analysts projects. They not only had well-specified questions, the teams contained internationally known experts in data analysis. While the teams lacked expertise in the domain topic, the questions provided were sufficiently specific to avoid error in this respect. The data itself, similarly, was well-suited to the questions and thus did not require expertise beyond that already contained in these teams.

The current ease of access to powerful analytical tools has led to many research projects lacking necessary expertise. Researchers are often tempted to rely on data analysis tools that can be easily found online or in software packages. This can be problematic if no one on the team understands how these methods work, what assumptions the methods make, what their results mean, and the best practices for using them. Even though applying these methods to data may be seemingly straightforward, developing Q* and selecting the correct approach for addressing it requires appropriate expertise.

#### Avoiding the pitfall

Ensure your team's expertise covers as many relevant skill and knowledge areas as possible, especially including the topic, data, and analysis method. You should also reassess what additional expertise may be required on your team after your Q* is identified.

## 4. Discussion

The recent surge of concern about variability in data analysis results stems from “many-analyst” papers, that empirically investigated what happens when multiple analysis teams are asked to solve the same problem, often with the same data set.

Data analysis contains challenges: there is an art to it, and this cannot be avoided by following a recipe. However, these challenges can be managed when project teams are aware of them. For example by avoiding the pitfalls described above. This contrasts the commonly observed attitude that data analysis simply has many subjective neither-right-nor-wrong elements constituting an unavoidable source of variation.

Conceptually, the emphasis here is on question formation (pitfall 1), data collection and preprocessing (pitfall 2), study design (pitfalls 2 and 3), and breadth and appropriate depth of expertise during study planning and execution (pitfall 3) as being principally responsible for study results. This is in contrast to choice of software package, analysis paradigm, or approach, which are commonly emphasized by others. Figure 1 reflects this focus: what many consider to be the core of data analysis is relegated to occupy only a fraction of the third step.

Study design, data collection, and question formation appear to be currently under-appreciated, at least among the published many-analyst papers and the communities that engage with them. These topics have received substantial attention previously (for example, see Kimball, 1957; Meehl, 1978; Hand, 1994; Hernán, 2016; Arnold and Franklin, 2021). Although these topics are receiving less attention today, modern computing, large quantities of convenient publicly available data, and convenient data analysis software packages have only exacerbated the problems. They are more important than ever before. Aside from the specific suggestions presented, the future of data analysis would also benefit from these topics receiving more attention in both educational and research settings.

## Data availability statement

Publicly available datasets were analyzed in this study. This data can be found here: https://osf.io/gvm2z/.

## Author contributions

EK and GJ contributed to the development, drafting, revisions, and basically everything. All authors contributed to the article and approved the submitted version.
